# Food Value of Two Varieties of Ginger (*Zingiber officinale*) Commonly Consumed in Nigeria

**DOI:** 10.5402/2013/359727

**Published:** 2013-01-17

**Authors:** Olubunmi B. Ajayi, Seun F. Akomolafe, Funmilayo T. Akinyemi

**Affiliations:** Department of Biochemistry, Ekiti State University, PMB 5363, Ado Ekiti, Nigeria

## Abstract

Ginger (*Zingiber officinale*) is a well-known and widely used herb, which contains several interesting bioactive constituents and possesses health-promoting properties. The proximate, mineral, antinutrient, amino acid, and phytochemical components of two varieties of ginger (*Zingiber officinale*) were investigated. Amino acid composition was determined using standard analytical techniques. The results obtained in percentages in the two varieties of ginger (white and yellow types) were crude fibre (21.90, 8.30), fat (17.11, 9.89), carbohydrate (39.70, 58.21), crude protein (12.05, 11.65), ash (4.95, 7.45) and moisture (3.95, 4.63) contents respectively. Elemental analysis revealed that potassium (0.98 ppm and 1.38 ppm) is the most abundant, while copper (0.01 ppm) is the least. Phytochemical screening indicated that they are both rich in saponins, anthraquinones, phlobatannin and glycosides. Also, the antinutrient constituents of white ginger were lower than yellow ginger, although the levels of the antinutrient constituents in the two varieties are saved for consumption. The essential amino acids in the two varieties were almost the same, with Leu being the most abundant in both. The two ginger varieties were adequate only in Leu, Phe + Try, and valine based on FAO/WHO provisional pattern. Overall, the findings indicate that the two varieties of ginger are good sources of nutrients, mineral elements, amino acid, and phytochemicals which could be exploited as great potentials for drugs and/or nutritional supplements.

## 1. Introduction

Plants such as herbs have long been used in traditional/folk medicine in various cultures throughout the world. *Zingiber officinale *is one of these traditional folk medicinal plants that have been used for over 2000 years for treating diabetes, high blood pressure, cancer, fitness, and many other illnesses [1]. Also, ginger (*Zingiber officinale*) is widely consumed as a spice and food preservation. The beneficial health effects of ginger have been well documented. According to Yoshikawa et al. [2], the consumption of ginger led to reduction in blood cholesterol and also served as a potential anti-inflammatory and antithrombotic agent.

Ginger is an herbaceous rhizomatous perennial plant that is widely cultivated in warm climatic regions of the world such as Nigeria, Bangladesh, Taiwan, India, Jamaica, and the United States of America. The rhizome contains a spectra of biologically active compounds such as curcumin, 6-gingerol, 6-shogoals, zingiberene, bisabolene, and several other types of lipids that confer on ginger the characteristics medicinal properties of being pungent and a stimulant [3, 4]. These properties have been reported to be responsible for its various medical applications as an analgesic, antiemetic, antiulcer, antipyretic, prostaglanding suppression, and cardio depressant among many others [5–7]. Ginger is added to a wide range of food as an indispensable curry powder or sauce. It is often used to flavour bread, tea, carbonated drinks, biscuits, pickles, and other confectionaries because of its aroma and flavour.

The objectives of the present study were to determine proximate composition, mineral element, amino acid profile, antinutrient constituents, and phytochemistry of two varieties of Nigerian gingers (*Zingiber officinale*), namely, white and yellow gingers.

## 2. Materials and Methods

### 2.1. Sample Collection and Preparation

Two different varieties of ginger (*Zingiber officinale*) were bought at the local market in Akungba Akoko, Ondo State, Nigeria. The plant materials were authenticated at the Department of Plant Science and Biotechnology, Adekunle Ajasin University, Akungba Akoko, Ondo State, Nigeria. The plants were washed, thinly sliced, oven dried at 60°C for 72 h, and milled. The powder obtained was stored in airtight plastic containers under refrigeration until needed for use.

### 2.2. Proximate and Mineral Analyses

The methods described in AOAC [8] were used to analyze the proximate composition of the plant for protein, fat, fibre, ash, and moisture, while carbohydrate was calculated by subtracting the sum of the values of the other nutrients from 100. The mineral analysis was carried out as described by Harbone [9] using Atomic Absorption Spectrophotometer (Pye Unican Sp9, Cambridge, UK). The minerals determined were sodium (Na), calcium (Ca), magnesium (Mg), potassium (K), phosphorus (P), iron (Fe), zinc (Zn), copper (Cu), and manganese (Mn).

### 2.3. Phytochemical Analysis

The phytochemical contents of the plants including saponins, tannins, anthraquinones, phlobatannin, cyanide, phytic acid, glycosides general, and glycosides with steroidal ring were qualitatively investigated [11, 12].

### 2.4. Amino Acid Analysis

Amino acid analysis was by Ion Exchange Chromatography (IEC) (FAO/WHO) [13], using the Technicon Sequential Multisample (TSM) amino acid analyzer (Technicon Instruments Corporation, NY, USA). The period of analysis was 76 mins for each sample. The gas flow rate was 0.50 mL/min at 60°C with reproducibility consistent within ±3%. The net height of each peak produced by the chart recorder of the TSM (each representing an amino acid) was measured and calculated. The amino acid values reported were the averages of two determinations.

Norleucine was the internal standard. Tryptophan was not determined.

The quality of dietary protein was measured by finding the ratio of available amino acids in the protein concentrate compared with needs expressed as a ratio [14]. Amino Acid Score (AMSS) was then estimated by applying the following FAO/WHO [13] formula:

(1) AMSS   =mg  of  amino  acid  in  1 g  test  protein  mg  of  amino  acid  in  1 g  of  reference  protein×1001.

## 3. Results and Discussion

The proximate compositions of two varieties of ginger (*Zingiber officinale*) white and yellow types were given in Table 1. The two varieties have low moisture content. The moisture contents of the white and yellow gingers were found to be 3.95, and 4.63%, respectively. The values compared favourably with the findings of Yusuf and Lasisi [15] for soybeans flour but lower than the value recorded by Akpata and Miachi [16] for *Dioclea reflexa* seeds flour. The difference in the moisture content could be explained by time interval between harvest and analysis, method of drying and storage, and the nature of the seed. This result implies that white ginger will have lower shelf life than yellow ginger. 

The protein content determination is one of the most important and widely used analytical measurements in processing and testing quality of food sample. The results obtained showed that white ginger has high protein content (12.05%) than yellow ginger (11.65%) but these values were lower when compared with those reported by Akpata and Miachi [16].

The two varieties contained appreciable amount of carbohydrates suggesting that they can be ranked as carbohydrate-rich spices (Table 1). However, the carbohydrate content of yellow ginger (58.21%) was higher than white ginger (39.70%), and these values were higher than what was reported by Ojimelukwe et al. [17].

White ginger showed high fat and crude fiber contents (17.11% and 21.9%, resp.) than yellow ginger (9.89% and 8.30%, resp.). The crude fibre content from the two samples was higher than some legumes such as cowpea [18], groundnut seed [19], and soyabean [20]. The ash content of yellow ginger was higher than white ginger.

The mineral analysis of the two varieties of ginger indicated their richness in calcium, magnesium, sodium, potassium, phosphorous, manganese, iron, zinc, and copper (Table 2). Potassium is the most abundant element found in both varieties. High amount of potassium in the body was reported to increase iron utilization [21], and it is beneficial to people taking diuretics to control hypertension and suffering from excessive excretion of potassium through the body fluid [22]. Both sodium and potassium are required to maintain osmotic balance of the body fluids, the pH of the body, to regulate muscle and nerve irritability, control glucose absorption, and enhance normal retention of protein during growth [23]. The ratio of sodium: potassium (Na : K) in the body is of great concern for the prevention of high blood pressure. A Na : K ratio of 1 is recommended [23]. Since this ratio is lower than 1 in both varieties, their consumptions would be beneficial to hypertensive patients. The level of K, P, Mg, and Na in yellow ginger was markedly higher than that of white ginger, while white ginger had a higher Ca content. However, the level of Zn, Cu, Fe, and Mn was lower in the two samples. The mineral values follow the orders K > P > Ca > Fe > Mg > Na > Mn > Zn > Cu and K > Ca > P > Fe > Na > Mg, Zn > Mn > Cu for yellow and white gingers, respectively.

The phytochemical analysis indicated that the two ginger varieties are rich in phytonutrients (Table 3). The result obtained revealed the presence of saponin, anthraquinones, phlobatannin, and glycosides.

The results of antinutritional factors of two varieties of ginger are presented in Table 4. Tannin value (0.26) and phytic acid value (28.88) of yellow ginger were higher than that of white ginger, while cyanide value (1.52) of white ginger was higher than that of yellow ginger. The antinutrients of yellow ginger were lower than those obtained in legumes [24] but similar to the reports of Adanlawo and Dairo [25] and Smith [26]. Even though the antinutrients are present in the two varieties of ginger, it may be for the defense of the stored reserves of food for the use of the plant [27, 28] and the level at which they occur in the two ginger varieties are safe for consumption by man and animals [25, 29].

Leucine was the most concentrated essential amino acid in the two ginger varieties; leucine content was higher in yellow ginger (56 mg/100 g protein) than in white ginger (42 mg/100 g protein) (Table 5). These values are in agreement with the observations made earlier by some researchers [30–32] suggesting that Leucine is the most concentrated essential amino acid in Nigerian plant foods. Glutamic acid was the most concentrated amino acid in the two varieties, also the value was higher in white ginger (56.80 mg/100 g protein) than in yellow ginger (35.80 mg/100 g protein). Tryptophan concentrations could not be determined.

The nutritive value of a protein depends primarily on the capacity to satisfy the needs for nitrogen and essential amino acids [33]. The total essential amino acid of yellow ginger (59.49%) was higher than white ginger (49.00%) (Table 6). The two values obtained are comparably similar with values obtained from soybeans [14] and some selected oil seeds [34] suggesting that the two ginger varieties can be used as food supplements. Essential Aliphatic Amino Acids (EAAAs), Ile, Leu, and Val, which constitute the hydrophobic regions of proteins were more abundant in yellow ginger (9.01 mg/100 g crude protein) than white ginger (7.47 mg/100 g crude protein) (Table 6). This means that better emulsification properties may be expected in the white ginger flour. Table 6 also depicts the percent of Total Acidic Amino Acids (TAAA) (20.76% and 26.68%) for yellow and white gingers, respectively, which were found to be greater than the percent of Total Basic Amino Acids (TBAA) (14.69% and 15.28%) for yellow and white gingers, respectively, indicating that the protein in the two varieties is probably acidic in nature. Total Sulphur Amino Acids (TSAAs) of the two varieties were 0.93 and 1.03 mg/100 g crude protein for yellow and white gingers, respectively. The TSAA for the two ginger varieties is lower than the 5.8 g/100 g crude protein recommended for infants [35]. These results confirm many works that have been reported ealiear on spices that were used as food additive for the purpose of flavour, medicine, colour, or as a preservative that kills harmful bacteria or prevent their growth [36, 37].

The contents of essential amino acid in the two varieties are generally lower than FAO/WHO [13] recommendations (Table 7). However, the contents of essential amino acid in the two varieties were almost the same. The two ginger varieties were adequate only in Leu, Phe + Try, and valine based on FAO/WHO provisional pattern, while they recorded low activities in some essential amino acids (Ile, met + cys, lys, and Thre). This result was consistent with the findings of [38]. Thus, based on our findings, the two ginger varieties may require supplementation with other rich sources in order to be used as confectionaries/or as a food supplement for any food material that is not adequate in essential amino acid. It has been reported that the essential amino acids most often acting in a limiting capacity are Met (and Cys), Lys, and Try [27]. However, the first three limiting amino acids in this study were yellow ginger (Ile, Met + Cys, and Lys) and white ginger (Thre, Ile, and Met + Cys) (Table 7). Try could not be determined.

## 4. Conclusion 

The present study has provided some biochemical information on the proximate composition, mineral element, amino acid profile, antinutrient constituents, and phytochemistry of two varieties of *Zingiber officinale *(white and yellow) commonly consumed in Nigeria. There are indications that the two varieties are good sources of nutrients, mineral elements, essential amino acids, and phytochemicals. Therefore, their use as nutritional supplements is highly promising.

## Figures and Tables

**Table 1:** Proximate composition of two varieties of ginger (*Zingiber officinale*).

Parameters type	Composition (%)	
	White type	Yellow type
Ash	4.95 ± 0.95	7.45 ± 0.45
Moisture	3.95 ± 2.70	4.63 ± 1.37
Fat	17.11 ± 7.10	9.89 ± 0.80
Crude fiber	21.90 ± 14.70	8.30 ± 6.90
Protein	12.05 ± 1.90	11.65 ± 0.45
Carbohydrate	39.70 ± 8.30	58.21 ± 6.50

Values are mean ± SD of 3 replicates.

**Table 2:** Mineral composition of two varieties of ginger (*Zingiber Officinale*).

Parameters	Composition (ppm)	
	White type	Yellow type
Ca	0.68 ± 0.01	0.41 ± 0.01
Mg	0.04 ± 0.01	0.11 ± 0.01
Na	0.26 ± 0.01	0.39 ± 0.01
K	0.98 ± 0.01	1.38 ± 0.01
P	0.42 ± 0.20	0.47 ± 0.01
Mn	0.03 ± 0.01	0.07 ± 0.04
Fe	0.29 ± 0.15	0.14 ± 0.01
Zn	0.04 ± 0.01	0.03 ± 0.01
Cu	0.01 ± 0.02	0.01 ± 0.06

Values are mean ± SD of 3 replicates.

**Table 3:** Phytochemical screening of two varieties of ginger (*Zingiber Officinale*).

Phytochemicals	White type	Yellow type
Saponin	+	+
Anthraquinones	+	+
Phlobatannin	+	+
Glycosides general	+	+
Glycosides with steroidal ring	+	+

+: present in moderate quantity; −: absent.

**Table 4:** Anti-nutrient compositions of two varieties of ginger (*Zingiber Officinale*).

Anti-nutrients	White type	Yellow type
Cyanide	1.520 ± 1.18	0.506 ± 0.17
Tannin	0.120 ± 0.05	0.260 ± 0.06
Phytic acid	20.18 ± 0.51	28.88 ± 0.73

Values are mean ± SD of 3 replicates.

**Table 5:** Amino acid composition of two varieties of ginger (*Zingiber Officinale*) (mg/100 g protein).

Amino acids	White type	Yellow type
Lysine^a^ (Lys)	2.70	15.90
Histidine (His)	10.40	5.00
Arginine^a^ (Arg)	41.40	26.80
Aspartic acid (Asp)	29.80	31.60
Threonine^a^ (Thre)	9.10	23.20
Serine (Ser)	23.10	10.20
Glutamic acid (Glu)	56.80	35.80
Proline (Pro)	15.00	8.10
Glycine (Gly)	22.60	17.10
Alanine (Ala)	10.60	9.90
Cystine (Cys)	4.60	4.60
Valine^a^ (Val)	22.00	23.70
Methionine^a^ (Met)	5.70	4.70
Isoleucine^a^ (Ile)	10.70	10.40
Leucine^a^ (Leu)	42.00	56.00
Tyrosine (Tyr)	11.10	14.20
Phenylalanine^a^ (Phe)	10.00	27.40

^a^Essential amino acids.

**Table 6:** Classification of amino acid composition (mg/100 g protein) of two varieties of ginger (*Zingiber Officinale*).

Amino acids	White type	Yellow type
Total amino acid (TAA)	36.08	32.46
Total nonessential amino acid (TNEAA)	18.40	13.15
% TNEAA	50.99	40.51
Total essential amino acid (TEAA)	17.68	19.31
% TEAA	49.00	59.49
Essential aliphatic amino acid (EAAA)	7.47	9.01
Essential aromatic amino acid (EArAA)	1.89	2.74
Total neutral amino acids (TNAA)	15.71	13.67
% TNAA	48.39	42.11
Total acidic amino acid (TAAA)	8.66	6.74
% TAAA	26.68	20.76
Total basic amino acid (TBAA)	5.88	4.77
% TBAA	15.28	14.69
Total sulphur amino acid (TSAA)	1.03	0.93
% Cysteine in TSAA	44.66	49.46

**Table 7:** Amino acid score of two varieties of ginger (*Zingiber Officinale*) (mg/g).

Essential amino acids	PAAESP^a^	Yellow type		White type	
		EAAC	AMSS	EAAC	AMSS
Isoleucine	40	10.40	0.26	10.70	0.27
leucine	70	56.00	0.80	42.00	0.60
Met + Cys	35	9.30	0.27	10.30	0.29
Lysine	55	15.90	0.29	27.00	0.49
Phe + Try	60	50.60	0.84	30.00	0.50
Threonine	40	23.20	0.58	9.10	0.23
Tryptophan	10	ND	ND	ND	ND
Valine	50	23.70	0.47	22.00	0.44

Total	360	189.10	3.51	151.10	2.82

^a^Source: Belschant et al. [10]. PAAESP: Provisional Amino Acid (Egg) Scoring Patterns; EAAC: Essential Amino Acid Composition (see Table 5); AMSS: Amino Acid Scores; ND: not determined.
